# Nomograms Predicting the Occurrence of Sepsis in Patients following Major Hepatobiliary and Pancreatic Surgery

**DOI:** 10.1155/2020/9761878

**Published:** 2020-08-01

**Authors:** Haoyun Zhang, Fanyu Meng, Shichun Lu

**Affiliations:** Department of Hepatobiliary Surgery, First Medical Center of Chinese People's Liberation Army (PLA) General Hospital, Chinese PLA Medical School, Beijing 100853, China

## Abstract

**Purpose:**

Sepsis is a severe complication in patients following major hepatobiliary and pancreatic surgery. The purpose of this study was to develop and validate a nomogram based on inflammation biomarkers and clinical characteristics.

**Methods:**

Patients who underwent major hepatobiliary and pancreatic surgery between June 2015 and April 2017 were retrospectively collected. Multivariate logistic regression was used to identify the independent risk factors associated with postoperative sepsis. A training cohort of 522 patients in an earlier period was used to develop the prediction models, and a validation cohort of 136 patients thereafter was used to validate the nomograms.

**Results:**

Sepsis developed in 55 of 522 patients of the training cohort and 19 of 136 patients in the validation cohort, respectively. In the training cohort, one nomogram based on clinical characteristics was developed. The clinical independent risk factors for postoperative sepsis include perioperative blood transfusion, diabetes, operative time, direct bilirubin, and BMI. Another nomogram was based on both clinical characteristics and inflammation biomarkers. Multivariate regression analyses showed that previous clinical risk factors, PCT, and CRP were independent risk factors for postoperative sepsis. The last nomogram showed a good *C*-index of 0.844 (95% CI, 0.787-0.900) compared with the previous one of 0.777 (95% CI, 0.713-0.840). Patients with a total score more than 109 in the second model are at high risk. The positive predictive value and negative predictive value of the second nomogram were 27% and 97%, respectively.

**Conclusion:**

The nomogram achieved good performances for predicting postoperative sepsis in patients by combining clinical and inflammation risk factors. This model can provide the early risk estimation of sepsis for patients following major hepatobiliary and pancreatic surgery.

## 1. Introduction

Severe sepsis and organ failure are major causes of morbidity and mortality after major hepatobiliary and pancreatic surgery [1]. Aggressive hepatobiliary pancreatic surgery has been associated with high complication rates of 40%–50% [1]. In a study of 583 patients who underwent surgical resection for intrahepatic cholangiocarcinoma, a postoperative complication occurred in 259 patients (44%) [2]. Moreover, sepsis is not uncommon. In one study of 1933 patients who underwent PD (pancreaticoduodenectomy), sepsis was found in 482 patients (24.9%) [3]. With advances in monitoring and prompt initiation of therapy, the mortality of severe sepsis remains higher than 25%~30%, even reaching 40-50% in the presence of shock [4]. These patients who survive to hospital discharge after sepsis are still at risk for death in the following time [5]. Those who survive often suffer from impaired physical or neurocognitive functions, emotional disorders, and a poor quality of life [6].

Sepsis was once defined as systemic inflammation caused by infection [7], and then, international consensus guidelines provide a series of warning signs of early sepsis [8]. Recently, it was defined as life-threatening organ dysfunction caused by dysregulated host response to infection [9]. Improper or delayed antibiotic treatment for sepsis patients can increase mortality [10]. Therefore, intravenous antibiotic therapy should be started as early as possible and should cover all likely pathogens [5]. This situation makes it meaningful to early predict the occurrence of sepsis.

In this study, we aim to develop and validate nomograms to predict sepsis of patients following major hepatobiliary and pancreatic surgery. Both serum biomarkers and clinical characteristics were analyzed by logistic regression analysis to identify risk factors. Then, one nomogram based on simple clinical characteristics and another one combining serum biomarkers and clinical characteristics were developed in a training cohort that comprises 522 patients. Finally, the nomograms were validated by a validation cohort containing 136 patients.

## 2. Materials and Methods

### 2.1. Patients

Patients who underwent major hepatobiliary and pancreatic surgery from June 2015 to April 2017 in the Department of Hepatobiliary Surgery of the First Medical Center of Chinese People's Liberation Army General Hospital were retrospectively studied. This study was approved by the Institutional Ethics Committee of Chinese People's Liberation Army General Hospital, and patients' informed consent was obtained. Patients in training and validation groups come from a 4 : 1 split of all patients by time and then were divided into the training and validation groups, respectively. Blood samples were collected 1 day after surgery and tested for procalcitonin (PCT) and C-reactive protein (CRP), cytokines, and peripheral neutrophil to lymphocyte ratio (NLR).

The main outcome was whether postoperative sepsis occurred in the included patients. The secondary outcomes included the time when sepsis occurred, various infection events such as pneumonia and biliary tract infection, and microbiological tests like body fluids and blood culture.

Patients with signs of infection and a SOFA score ≥ 2 points were identified as sepsis [9]. In clinical use, patients with suspected and culture-proven infection are both in consideration. The SOFA comprises a score relevant to the major organ systems and graded from 0 to 4 according to the degree of dysfunction or failure [11].

Major surgery which was defined as organ removal or normal surgical anatomy which has changed after peritoneal access as previously reported, including operations creating any gastrointestinal anastomosis or involving parenchymal resection of the liver, bile duct, spleen, or pancreas, were included [12, 13]. In this study, we mainly included hepatectomy, pancreaticoduodenectomy, hilar cholangiocarcinoma radical surgery, and other hepatobiliary operations, and less extensive surgery such as cholecystectomy and liver tumor radiofrequency ablation was excluded. But surgery including both hepatectomy and cholecystectomy was included.

The exclusion criteria were patients with age < 18, with liver radiofrequency ablation only, with laparoscopic cholecystectomy only, and with incomplete clinical and inflammation biomarker data.

### 2.2. Methods

In this study, we collected patients' clinical characteristic data on gender, age, body mass index (BMI), and diabetes. Perioperative data included durations of anesthesia, operative time, ASA physical status score, hemorrhage, and blood transfusion volumes. Perioperative blood transfusion in this study was based on data from 3 days after surgery and before.

Laboratory tests include direct bilirubin (DBil), aspartate aminotransferase (AST), alanine aminotransferase (ALT), lymphocyte counts, neutrophil counts, white blood cell count (WBC), C-reactive protein (CRP), procalcitonin (PCT), results of blood bacterial culture, and identified bacteria. Serum biomarkers (IL-1, IL-2, IL-6, IL-8, IL-10, and TNF-*α*) and peripheral neutrophil to lymphocyte ratio (NLR) were detected at 1 day after surgery.

Two surgeons separately collected clinical and inflammatory biomarker data of all patients who met the inclusion criteria. For inconsistencies in data, final results were confirmed by carefully reviewing the electronic medical records.

Serum IL-1, IL-2, IL-6, IL-8, IL-10, and TNF-*α* were measured by chemiluminescent immunoassay technology on the IMMULITE 1000 Immunoassay System as previously reported [14]. IL-6 was measured by the IMMULITE 2000 Immunoassay System.

### 2.3. Statistical Analysis

Normally distributed data of continuous variables were expressed as the mean ± standard deviation and compared by an unpaired, 2-tailed *t-*test. Otherwise, the data were represented as the median (P25 and P75) and compared by the Mann-Whitney test. Categorical variables were compared by the chi-squared test or Fisher exact test. Univariate logistic regression was performed in the training cohort to identify variables that were associated with postoperative sepsis, and then, multivariate logistic regression analysis was used to determine whether the variables included were independent.

Nomograms were built on multivariate logistic regression results using the “rms” package of R. One nomogram based on clinical characteristics and another one based on both clinical characteristics and inflammation biomarkers were developed. The predictive performance of the 2 nomograms in the training cohort was measured by *C*-index and with 1000 bootstrap samples to decrease the overfit bias. Then, the predicted probabilities of the 2 models in the validation cohort were shown as ROC curves.

For clinical use, the total scores of each patient were calculated based on the nomograms. The cutoff value for total points of postoperative sepsis was calculated using the “OptimalCutpoints” package of R based on maximizing the Youden index. The net reclassification improvement (NRI) was calculated using the “PredictABEL” package of R. *P* < 0.05 was considered to indicate statistical significance. All analyses were performed in SPSS and R, version 3.6.1.

## 3. Results

### 3.1. Clinical Characteristics and Inflammation Biomarkers

A total of 658 patients who underwent major hepatobiliary and pancreatic surgery and met the inclusion criteria were included. 522 patients and 136 patients were divided into the training and validation groups, respectively.

The inflammation biomarkers were detected at 1 day after surgery. Patients' clinical characteristics in the training and validation cohorts were given in Table 1. There were no significant differences between the 2 cohorts in sepsis prevalence (*P* = 0.26). The finally diagnosed sepsis was found in 55 and 19 patients in the training and validation cohorts, respectively. Tables 2 and 3 show the characteristics of sepsis and nonseptic patients in the training and validation cohorts, respectively.

All patients in this study underwent postoperative (one day postsurgery) blood test for PCT, CRP, and cytokines (IL-1, IL-2, IL-6, IL-8, IL-10, and TNF-*α*). NLR was also collected. The median levels and interquartile ranges of inflammation biomarkers are listed in Table 4. There were no significant differences among the 2 cohorts.

### 3.2. Development and Validation of Nomograms

The results of univariate logistic regression analysis are presented in Table 5. The results were reported as odds ratio (95% CI). Two different multivariate logistic regression analyses were performed. The first one only included clinical characteristics as shown in Table 6, and the second one included both clinical characteristics and inflammation biomarkers as shown in Table 7. In the first regression results, perioperative blood transfusion (OR: 3.638 (1.921-6.891)), diabetes (OR: 2.378 (1.221-4.633)), operative time (OR: 1.003 (1.001-1.005)), direct bilirubin (OR: 1.004 (1.001-1.007)), and BMI (OR: 1.164 (1.057-1.281)) were independently associated with sepsis. In the second regression results, these clinical characteristics plus PCT (OR: 1.143 (1.071-1.221)) and CRP (OR: 1.175 (1.081-1.277)) were independently associated with sepsis.

**Figure 1:**
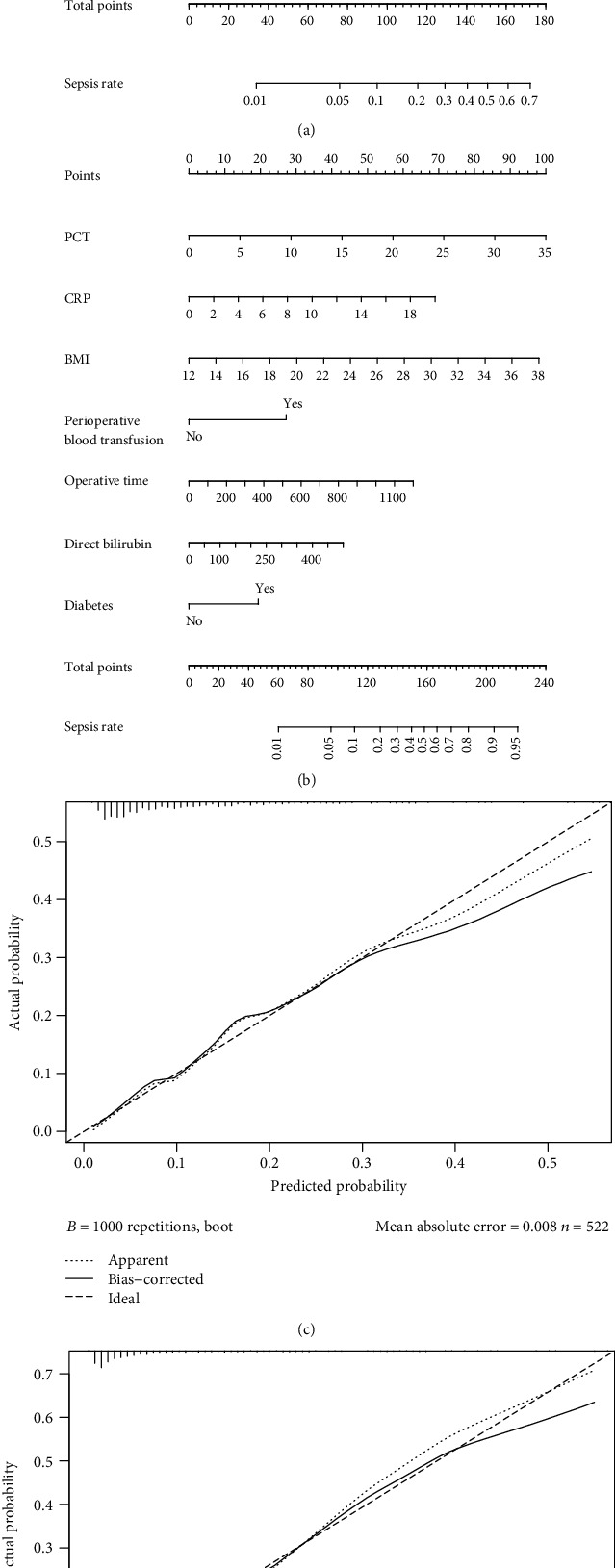
(a) Nomogram based on clinical variables for predicting sepsis in patients following major hepatobiliary and pancreatic surgery. (b) Nomogram based on clinical and inflammation variables for predicting sepsis in patients following major hepatobiliary and pancreatic surgery. (c) Bootstrap validation of the predictive performance of the first nomogram in estimating the risk of sepsis in the training cohort. (d) Bootstrap validation of the predictive performance of the second nomogram in estimating the risk of sepsis in the training cohort.

Sepsis risk estimation nomograms based on the 2 multivariate regression analysis results were developed as shown in Figure 1. Both nomograms showed good accuracy in predicting sepsis. The 2 nomograms were validated using the bootstrap method in the training cohort. The first one has a *C*-index of 0.777 (95% CI, 0.713-0.840) and a bootstrap-corrected *C*-index of 0.761, and the second one has a *C*-index of 0.844 (95% CI, 0.787-0.900) and a bootstrap-corrected *C*-index of 0.831.

### 3.3. Compare the Performance of Nomograms for Predicting Sepsis in the Validation Cohort

**Figure 2:**
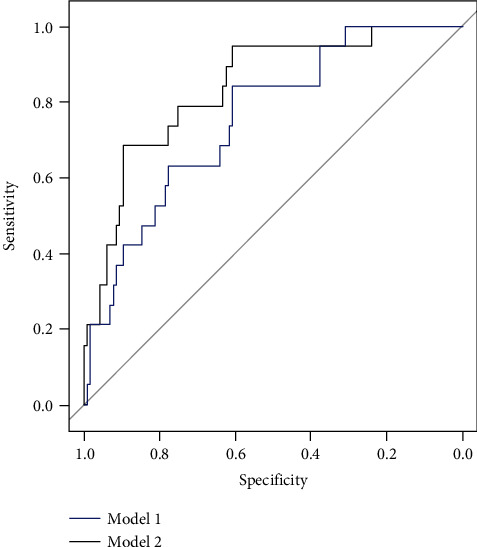
“Model 1” showed the diagnostic performance of predicted probabilities in the validation cohort based on clinical variables; “model 2” showed the diagnostic performance of predicted probabilities in the validation cohort based on clinical variables and inflammation biomarkers.

The predicted sepsis probabilities of the 2 different nomograms for patients in the validation cohort were calculated. The diagnostic performances of the 2 different models for the validation cohort were evaluated by ROC curves and shown in Figure 2. In the validation cohort, the first nomogram showed an AUC of 0.756 (95% CI, 0.647-0.864); the second nomogram showed an AUC of 0.839 (95% CI, 0.745-0.932). There is a significant difference between these two ROC curves, with a *P* value of 0.048. Besides, we could see that the AUC of model 2 was larger.

### 3.4. Cutoff Value of the Predicting Risk for Estimating Sepsis

To better use these nomograms for patients, the best cutoff total scores for risk estimation of the 2 nomograms were calculated based on maximizing the Youden index. We found that at cutoff total scores of 106 for the first model and 109 for the second model, respectively, these two prediction models have the largest Youden index as shown in Table 8. Using 106 for the first model and 109 for the second model as our cutoff values for high risk, the positive predictive value was 33% (validation cohort) and the negative predictive value was 91% (validation cohort) for model 1; the positive predictive value was 35% (validation cohort) and the negative predictive value was 95% (validation cohort) for model 2. The positive likelihood ratio and negative likelihood ratio in the training or validation cohort for both models are also shown in Table 8.

To demonstrate the superiority of PCT and CRP in model 2, we then compare the performance of the 2 models for the validation cohort, and the net reclassification improvement (NRI) was 0.1125 (*P* = 0.43).

## 4. Discussion

In this study, we developed and validated 2 different nomograms for early prediction of sepsis in patients following major hepatobiliary and pancreatic surgery. The last nomogram incorporates 2 inflammation biomarkers including the serum level of PCT and CRP. Incorporating the inflammation biomarkers and clinical risk factors into nomograms facilitates the early prediction of sepsis.

Similar to our results, a meta-analysis of over 30 million patients identified that the risk factors associated with postoperative sepsis also include perioperative blood transfusion (OR: 1.90) and diabetes (OR: 1.41) [15]. Other risk factors reported include male gender and emergency surgery [16]. Most of these studies were based on patient and surgery-related risk factors for postoperative sepsis. In our study, inflammation biomarkers like cytokines (IL-1, IL-2, IL-6, IL-8, IL-10, and TNF-*α*), PCT, CRP, and NLR were studied. IL-2, IL-6, IL-10, and NLR were significant after univariate regression analysis, but they were not independent risk factors for sepsis in our study.

PCT and CRP are both widely used in diagnosis of sepsis [17]. PCT is a peptide released in response to proinflammatory stimuli, particularly bacterial-related inflammatory mediators [18], although there is disagreement on the accuracy of PCT for differentiating sepsis from other noninfectious causes of SIRS (systemic inflammatory response syndrome) [19]. In this study, we found that PCT (*P* ≤ 0.001, 1.143 (1.071-1.221)) was an independent risk factor for the occurrence of sepsis among patients who underwent major hepatobiliary and pancreatic surgery. No better markers are available that outperform PCT in diagnosis of sepsis [20, 21]. CRP showed significant differences in both training (*P* ≤ 0.001) and validation (*P* ≤ 0.001) cohorts. In multivariate analysis, CRP (*P* ≤ 0.001, OR: 1.175 (1.081-1.277)) served as an independent risk factor. It was reported that an elevated serum CRP level is correlated with increased risk of organ failure and death [22].

Regarding clinical risk factors, preoperative direct bilirubin levels are independent risk factors for sepsis. It was reported that jaundice (total bilirubin > 2.5 mg/dl) was associated with 30-day mortality in patients with bacteraemic cholangitis [23]. Patients with biliary obstruction are common in hepatobiliary surgery, and much attention should be paid to these patients. BMI was also independent risk factors for sepsis in our study; BMI was reported to be associated with increased risk of infections [24]. Obese patients with BMI > 30 kg/m^2^ should be noted. In our study, operative time was also an independent risk factor for sepsis. Duration of operation was reported to correlate with complications, and long duration procedures had greater risk of sepsis/sepsis shock [25]. Blood transfusion was also reported to be independently associated with higher odds of sepsis and septic shock and increased overall 30-day mortality [3]. In our study, blood transfusion was an independent risk factor for the occurrence of sepsis.

As shown in Figure 2, the last nomogram showed a bigger AUC for the validation group. And the 2 nomograms showed significant differences in predicting the sepsis risk of the validation cohort. The addition of inflammation biomarkers into the predictive model obtained satisfactory improvement for sepsis prediction (the *C*-index of model 1 was 0.777 in the training cohort and 0.756 in the validation cohort; the *C*-index of model 2 was 0.844 in the training cohort and 0.839 in the validation cohort). For clinical use of the 2 models, we evaluate the sensitivity, specificity, positive predictive value, and negative predictive value in risk estimation of sepsis using the method of Youden index. Patients with total scores more than 109 in model 2 are at a high-risk subgroup of sepsis. A high negative predictive value (97% in the training cohort) was yielded, but the positive predictive value (27% in the training cohort) was less impressive. Considering that the cost for early interventions such as the timely use of antibiotics and the improvement of bacterial culture tests is low and the patients will get the maximum benefit, we believe that the last model will allow surgeons to estimate the sepsis risk of patients who underwent major hepatobiliary and pancreatic surgery soon after surgery, and corrective treatment can be applied in time.

A limitation of our study mainly includes its retrospective design and a relatively smaller sample size. Patients in training and validation groups come from a split of all patients by time in our institution rather than an independent group of patients. Therefore, more strict external validation and prospective study are needed.

## 5. Conclusion

By combining clinical and inflammatory risk factors, we constructed a nomogram for postoperative sepsis for patients following major hepatobiliary and pancreatic surgery. This model can provide the early risk estimation of sepsis for patients after major hepatobiliary and pancreatic surgery.

## Figures and Tables

**Table 1:** Characteristics of patients in the training and validation cohorts.

Variables	Cohort		*P* value
	Training (*n* = 522)	Validation (*n* = 136)	
Age	55.82 ± 12.91	56.60 ± 11.11	0.52
Gender			
Male	339	97	0.16
Female	183	39	
Diabetes			
Yes	95	20	0.34
No	427	116	
BMI	23.62 ± 3.22	23.55 ± 3.73	0.83
Perioperative blood transfusion			
Yes	203	45	0.21
No	319	91	
Preoperative ALT (U/l)	45.05 (21.52-121)	40.5 (20.2-117.2)	0.47
Preoperative AST (U/l)	37.55 (21.2-96.75)	36.7 (19.57-79.45)	0.48
Preoperative direct bilirubin (*μ*mol/l)	5.4 (3.3-38.8)	5.25 (3.2-29.43)	0.83
Intraoperative blood loss (ml)	300 (200-600)	300 (200-500)	0.51
Operative time (min)	285 (200-345)	285 (200-365)	0.75
ASA score			
1	11	4	0.14
2	434	107	
3	75	22	
4	2	2	
5	0	1	

**Table 2:** Clinical characteristics and inflammation biomarkers in the training cohort. Data are shown as the mean ± SD or median (P25 and P75).

Variable	Training cohort		
	Sepsis (-), *n* = 467	Sepsis (+), *n* = 55	
Age	55.41 ± 13.05	59.29 ± 11.24	0.02^∗^
Sex			
Male	305	34	0.61
Female	162	21	
Perioperative blood transfusion			
Yes	165	38	≤0.001^∗^
No	302	17	
ASA scores			
1	10	1	≤0.001^∗^
2	392	42	
3	64	11	
4	1	1	
Diabetes			
Yes	78	17	0.01^∗^
No	389	38	
BMI	23.5 ± 3.21	24.69 ± 3.12	0.01^∗^
Operative time (min)	280 (210-340)	345 (262.5-431)	0.03^∗^
Intraoperative blood loss	300 (200-600)	400 (200-800)	
Preoperative ALT	44 (20.45-122.5)	62 (24.5-104.9)	0.53
Preoperative AST	35.6 (20.85-98.95)	42.6 (26.4-81.9)	0.47
Preoperative direct bilirubin	5.1 (3.2-31)	35.5 (5.1-132.05)	≤0.001^∗^
Inflammation biomarkers			
IL-1 (pg/ml)	5 (5-5)	5 (5-5)	0.46
IL-2 (U/ml)	767 (600-1023)	1034 (699.5-1378.5)	≤0.001^∗^
IL-6 (pg/ml)	82.2 (46.15-164.5)	171 (102-274)	≤0.001^∗^
IL-8 (pg/ml)	89 (40.2-190)	105 (55.55-242)	0.18
IL-10 (pg/ml)	5.78 (5-10)	9.56 (5.86-14.35)	≤0.001^∗^
TNF-*α* (pg/ml)	16.3 (11-30.55)	16.5 (10.5-24.95)	0.50
PCT (ng/ml)	0.55 (0.28-1.01)	1.53 (0.809-3.29)	≤0.001^∗^
CRP (mg/dl)	3.73 (1.96-6.29)	6.62 (4.62-9.35)	≤0.001^∗^
NLR	11.1 (7.73-15.73)	12.17 (8.10-16.94)	0.39

**Table 3:** Clinical characteristics and inflammation biomarkers of patients in the validation cohort. Data are shown as the mean ± SD or median (P25 and P75).

Variable	Validation cohort		
	Sepsis (-), *n* = 117	Sepsis (+), *n* = 19	
Age	55.8 ± 11.07	61.53 ± 10.30	0.04^∗^
Sex			
Male	83	14	0.81
Female	34	5	
Perioperative blood transfusion			
Yes	33	12	≤0.001^∗^
No	84	7	
ASA scores			
1	4	0	0.04^∗^
2	95	12	
3	17	5	
4	1	1	
5	0	1	
Diabetes			
Yes	15	5	0.16
No	102	14	
BMI	23.47 ± 3.77	24.02 ± 3.54	0.56
Operative time	265 (190-345)	395 (330-485)	≤0.001^∗^
Intraoperative blood loss	300 (150-500)	300 (300-500)	0.10
Preoperative ALT	39.5 (19.6-116.6)	57 (26.9-117.5)	0.52
Preoperative AST	35 (20-79.1)	47.6 (17.9-104.85)	0.77
Preoperative direct bilirubin	4.6 (3.2-14.9)	14.8 (5.05-144.75)	0.03^∗^
Inflammation biomarkers			
IL-1 (pg/ml)	5 (5-5)	5 (5-5)	0.31
IL-2 (U/ml)	805 (591-1053)	1226 (978-1963)	≤0.001^∗^
IL-6 (pg/ml)	64.6 (37.5-154)	151 (97.75-350.5)	0.01^∗^
IL-8 (pg/ml)	96 (36.9-187)	112 (81.95-231.5)	0.27
IL-10 (pg/ml)	5 (5-5)	6.57 (5-11.5)	0.25
TNF-*α* (pg/ml)	14 (10.2-25.8)	22.2 (17.8-35.25)	0.02^∗^
PCT (ng/ml)	0.544 (0.26-0.911)	1.23 (0.89-2.43)	≤0.001^∗^
CRP (mg/dl)	3.28 (1.75-5.18)	5.93 (3.88-13.1)	≤0.001^∗^
NLR	10.96 (7.87-16.96)	9.51 (4.72-12.49)	0.18

^∗^ *P* < 0.05.

**Table 4:** The median levels and interquartile ranges of inflammation biomarkers in the training and validation cohorts.

Variables	Cohort		*P* value
	Training (*n* = 522)	Validation (*n* = 136)	
IL-1 (pg/ml)	5 (5-5)	5 (5-5)	0.75
IL-2 (U/ml)	788 (607.5-1074.8)	851 (625-1106)	0.33
IL-6 (pg/ml)	90.55 (48.12-178.5)	70.7 (39.52-191.25)	0.20
IL-8 (pg/ml)	91.15 (41.95-194.5)	99 (38.08-188)	0.90
IL-10 (pg/ml)	6.02 (5-10.38)	6.33 (5-10.4)	0.66
TNF-*α* (pg/ml)	16.35 (11-29.9)	15.75 (10.3-28.45)	0.41
PCT (ng/ml)	0.60 (0.32-1.14)	0.57 (0.31-1.10)	0.90
CRP (mg/dl)	4.02 (2.14-6.71)	3.65 (2.03-5.41)	0.13
NLR	11.22 (7.76-15.85)	10.88 (7.62-16.66)	0.63

**Table 5:** Univariate regression analysis of sepsis based on clinical characteristics and inflammation biomarkers in patients following major surgery.

Variables	OR	*P*
IL-1 (pg/ml)	0.997 (0.963-1.032)	0.862
IL-2 (U/ml)	1.001 (1.000-1.001)	0.001^∗^
IL-6 (pg/ml)	1.003 (1.001-1.004)	≤0.001^∗^
IL-8 (pg/ml)	1.000 (1.000-1.001)	0.125
IL-10 (pg/ml)	1.005 (1.001-1.009)	0.016^∗^
TNF-*α* (pg/ml)	1.001 (0.994-1.008)	0.697
PCT (ng/ml)	1.134 (1.066-1.205)	≤0.001^∗^
CRP (mg/dl)	1.209 (1.125-1.300)	≤0.001^∗^
NLR	1.030 (1.002-1.057)	0.033^∗^
Perioperative blood transfusion (yes vs. no)	4.091 (2.240-7.474)	≤0.001^∗^
ASA scores	1.715 (0.920-3.197)	0.090
Diabetes (yes vs. no)	2.231 (1.198-4.153)	0.011^∗^
BMI	1.12 (1.027-1.221)	0.01^∗^
Age	1.026 (1.002-1.050)	0.036^∗^
Gender (male vs. female)	0.86 (0.483-1.53)	0.608
Operative time	1.003 (1.002-1.005)	≤0.001^∗^
Intraoperative blood loss	1.000 (1.000-1.001)	0.013^∗^
ALT (U/l)	1.001 (0.999-1.002)	0.278
AST (U/l)	1.001 (1.000-1.002)	0.17
Direct bilirubin (*μ*mol/l)	1.005 (1.002-1.008)	0.001^∗^

ASA: American Society of Anesthesiologists. ^∗^*P* < 0.05.

**Table 6:** Multivariate regression analysis of sepsis based on clinical characteristics in patients following major surgery.

Variable	*B*	*P*	OR (95% CI)
Blood transfusion (yes vs. no)	1.291	≤0.001	3.638 (1.921-6.891)
Diabetes (yes vs. no)	0.866	0.011	2.378 (1.221-4.633)
Operative time (min)	0.003	0.012	1.003 (1.001-1.005)
Preoperative direct bilirubin (*μ*mol/l)	0.004	0.010	1.004 (1.001-1.007)
BMI	0.151	0.002	1.164 (1.057-1.281)

**Table 7:** Multivariate regression analysis of sepsis based on clinical characteristics and inflammation biomarkers in patients following major surgery.

Variable	*B*	*P*	OR (95% CI)
Blood transfusion (yes vs. no)	1.276	≤0.001	3.583 (1.815-7.072)
Diabetes (yes vs. no)	0.910	0.012	2.485 (1.224-5.046)
Operative time (min)	0.002	0.023	1.002 (1.000-1.005)
Preoperative direct bilirubin (*μ*mol/l)	0.004	0.019	1.004 (1.001-1.007)
BMI	0.177	0.001	1.193 (1.075-1.325)
PCT (ng/ml)	0.134	≤0.001	1.143 (1.071-1.221)
CRP (mg/dl)	0.162	≤0.001	1.175 (1.081-1.277)

**Table 8:** Diagnostic performances of the 2 nomograms for postoperative sepsis estimation.

Variables	Training cohort	Validation cohort
Model 1 (clinical variable)		
Cutoff score	106	106
Sensitivity	0.64	0.47
Specificity	0.82	0.85
Positive predictive value	0.29	0.33
Negative predictive value	0.95	0.91
Positive likelihood ratio	3.50	3.13
Negative likelihood ratio	0.44	0.62
Model 2 (clinical and inflammation variables)		
Cutoff score	109	109
Sensitivity	0.84	0.74
Specificity	0.73	0.78
Positive predictive value	0.27	0.35
Negative predictive value	0.97	0.95
Positive likelihood ratio	3.15	3.36
Negative likelihood ratio	0.22	0.33

## Data Availability

The data used to support the findings of this study are available from the corresponding author upon request.
